# ﻿The solitary wasp genus *Orancistrocerus* from Vietnam, with descriptions of two new species (Hymenoptera, Vespidae, Eumeninae)

**DOI:** 10.3897/zookeys.1193.116087

**Published:** 2024-03-01

**Authors:** Lien Thi Phuong Nguyen, Anh D. Nguyen, Hoa T. Dang

**Affiliations:** 1 Insect Ecology Department, Institute of Ecology & Biological Resources (IEBR), Vietnam Academy of Science & Technology, 18 Hoang Quoc Viet Road, Nghia Do, Cau Giay, Hanoi, Vietnam Institute of Ecology & Biological Resources (IEBR), Vietnam Academy of Science & Technology Hanoi Vietnam; 2 Graduate University of Science and Technology, Vietnam Academy of Science & Technology, 18 Hoang Quoc Viet Road, Nghia Do, Cau Giay, Hanoi, Vietnam Graduate University of Science and Technology Hanoi Vietnam

**Keywords:** Biodiversity, new description, Oriental region, potter wasps, synonym, taxonomy, Vietnam

## Abstract

New data are presented for the potter wasp genus *Orancistrocerus* van der Vecht (Eumeninae, Odynerini) occurring in Vietnam. Two species are described as new to science: *Orancistrocerusthanhnhat***sp. nov.** and *O.thanghen***sp. nov.***Orancistrocerusaterrimuserythropus* van der Vecht is synonymized with *Orancistrocerusaterrimusaterrimus* (de Saussure); the male genitalia of this species are described for the first time. An updated key is presented to all species of the genus.

## ﻿Introduction

Truong et al. (2019) explored the diversity and systematics of the little-known potter wasp genus *Orancistrocerus* van der Vecht, 1963 from Vietnam, and they described *Orancistrocerusaltus* Truong, Bozdogan, & Nguyen, 2019, thereby raising the number of known species in the genus to five: *O.aterrimus* (de Saussure, 1852), *O.drewseni* (de Saussure, 1857, *O.bicoloripennis* (Gribodo, 1892), *O.moelleri* (Bingham, 1897), and *O.altus*. These species are distributed in Eastern Asia, from China and Japan south to Borneo.

During several recent field expeditions to Cao Bang Province, with support from the Vietnam Academy of Science and Technology under the project, “Developing the first-class research team on the discovery of diversity and application potential of hymenopterans, myriapods, and soil nematodes in the limestone mountains of northeastern Vietnam”, specimens of two undescribed species of *Orancistrocerus* were collected. Based on the material deposited in the Insect Ecology Department, Institute of Ecology and Biological Resources, Hanoi, Vietnam, a new taxonomic study was undertaken on *Orancistrocerus* from Vietnam. The results of that work are presented here, with descriptions and figures of two new species. Additionally, an updated key is presented to all known species in the genus.

## ﻿Materials and methods

Specimens of *Orancistrocerus* from the Insect Ecology Department, Institute of Ecology and Biological Resources (**IEBR**), Hanoi, Vietnam were examined. Morphological and color characters of mature specimens were observed using pinned and dried specimens under an Olympus SZ4 stereomicroscope, and measurements were made with an ocular micrometer. “Body length” indicates the combined lengths of the head, mesosoma, and the first two metasomal segments. Morphological terminology follows that of Carpenter and Cumming (1985) and Yamane (1990). Genitalic terminology follows Kojima (1999) and Nguyen et al. (2023). Photographic images were made with a Nikon SMZ 800N Digital Stereo Microscope and an attached Sony α6000 digital camera. Images were stacked using Helicon Focus v. 7, then grouped into a figure using Adobe Photoshop CS6. The abbreviations **F**, **S**, and **T** (I, II, III, …) refer to numbered flagellomeres, metasomal sterna, and metasomal terga, respectively. Other abbreviations are: **NP**, National Park; **NR**, National Reserve; **ISD-c**, collectors from the Insect Systematic Department (IEBR).

## ﻿Systematics

### ﻿Genus Orancistrocerus

Taxon classificationAnimaliaHymenopteraVespidae

van der Vecht, 1963

905B6665-D9D3-5053-A339-2B5E50FBC5FC

Orancistrocerus van der Vecht, 1963: 58, 99. Type species: Odynerusdrewseni de Saussure, 1857, by original designation.

#### Diagnosis.

Anterior surface of pronotum without pits or foveae. Tegula not evenly rounded posteriorly, emarginate adjoining parategula and often shorter than the latter. Axillary fossa in dorsal view much narrower than long, slit-like. Propodeum without deep fossae, submarginal carina and valvula not protruding; propodeal dorsum not forming raised shelf-like area behind metanotum. Metasomal terga with short apical lamellae; TI transversely carinate, without broad longitudinal median furrow posterior to carina, long, dorsal surface ~2× or less as wide as long. Male antenna hooked apically.

#### Generic relationships.

*Orancistrocerus* is a small genus, currently with five species and with four valid subspecies in *O.aterrimus*, which is widely distributed from India to China, including Laos and Vietnam. The second most widely distributed species, *O.drewseni*, with three subspecies, is distributed in several tropical climatic zones of China (including Taiwan), Japan, and Laos, while the two subspecies of *O.bicoloripennis* is distributed only in Malaysia and Indonesia. *Orancistrocerusmoelleri*, with two subspecies, is recorded from South China, northern India adjacent to South China, and Myanmar (van der Vecht 1963; Giordani Soika 1973; Tan et al. 2018). The remaining species, *O.altus*, *O.thanghen* sp. nov., and *O.thanhnhat* sp. nov., are known only from northern Vietnam, but it is assumed that these also occur in southern China as the type localities border that region. More interestingly, all three Vietnamese species have been found only in limestone areas.

Van der Vecht (1963) established the genus *Orancistrocerus* but did not discuss any similarities or relationships between *Orancistrocerus* and other eumenine genera. Recently, a phylogenetic tree showing relationships within the subfamily Eumeninae was provided based on molecular data (Luo et al. 2022), and in that study, *Orancistrocerus* is closely related to *Euodynerus*. Morphological similarities of these two genera are as follows: metasoma not petiolate; fore wing with second submarginal cell not petiolate; propodeum without deep fossae; metanotum without tubercles; anterior surface of pronotum without pits or foveae. TI transversely carinate is a character to separate *Orancistrocerus* from *Euodynerus* (TI not carinate in *Euodynerus*). In the phylogenetic tree by Luo et al. (2022), *Orancistrocerus* and *Pararrhynchium* are not closely related, but they are very similar to each other in morphology, owing to the metasoma not petiolate, forewing with second submarginal cell not petiolate, metanotum without tubercles, anterior face of pronotum without pits or foveae, propodeum with submarginal carina and valvula not produced, TI transversely carinate, axillary fossa narrower than long, slit-like, tegula not exceeding parategula, and male antenna hooked apically. *Orancistrocerus* differs from *Pararrhynchium* in having the propodeal dorsum not forming a shelf-like area behind the metanotum and the metasomal terga with short apical borders (propodeal dorsum usually forming a shelf-like area behind the metanotum more than one ocellar diameter long; metasomal terga usually with some well-developed apical lamellae in *Pararrhynchium*). The genus *Malayepipona* was not included by Luo et al. (2022), but, based on a study of the Vietnamese material, *Orancistrocerus* is morphologically also similar to *Malayepipona* in the shape of the mesosoma, propodeum, and metasoma in dorsal view, in having the first metasomal tergum angular in profile, and TI with the anterior portion separated from the horizontal part by a more or less distinct transverse ridge. These two genera can be distinguished in having the tegula not exceeding the parategula, axillary fossa in dorsal view much narrower than long, slit-like in *Orancistrocerus* (tegula at least equaling parategula posteriorly; axillary fossa in dorsal view not slit-like, oval in *Malayepipona*).

#### Origin of genus.

Even if there is no existing hypothesis on biogeographic history of *Orancistrocerus*, our working hypothesis is that these species may have originated in the Himalayas running between China and India, perhaps during episodes of orogeny, then dispersed into South China and northern India as well as Southeast Asia, including Vietnam, Myanmar, Malaysia, and Indonesia. Naturally, a comprehensive revision and phylogenetic analysis are needed to test this hypothesis and establish a robust estimate for the complex biogeographic history of *Orancistrocerus* and other Asiatic Eumeninae.

### ﻿ Orancistrocerus thanghen sp. nov.

Taxon classificationAnimaliaHymenopteraVespidae

3029D3C9-BF58-5AC0-AFFC-7E2AF9E5D077

https://zoobank.org/C6CA3009-D439-41A3-8A14-0A8C974E964C

Fig. 1A–G

#### Material examined.

***Holotype*.** Vietnam • ♀; Cao Bang, Tra Linh, Thang Hen lake; 22°45'48"N, 106°17'38"E; alt. 611 m; 3.viii.2022; Lien Thi Phuong Nguyen, Cuong Quang Nguyen, Ngat Thi Tran leg.; IEBR.

***Paratypes*.** Vietnam • 4♀♀, same data as holotype • 1♀; Cao Bang, Tra Linh, Thang Hen Lake; 22°45'47.5"N, 106°17'35.7"E; alt. 619 m; 18.xi.2023; Lien Thi Phuong Nguyen, Duc Anh Nguyen, Ngat Thi Tran leg.; IEBR.

#### Diagnosis.

This species can be distinguished from other species in the genus in the following combination of characters: head in frontal view subcircular, 1.1× as wide as high; occipital carina slightly widened laterally; clypeus in frontal view 1.2× as wide as high, apical margin deeply emarginated medially, forming blunt tooth on each side, distance between teeth ~0.5× width of clypeus between inner compound eye margins; mesoscutum shorter than wide between tegulae; TI in dorsal view ~2.2× as wide as long; TII densely punctate, interspaces larger (~1.5×) than puncture diameter.

The new species is similar to *O.altus* in that both have TII densely punctate, interspaces larger (~1.0–1.5×) than puncture diameter, the occipital carina slightly widened laterally, mesoscutum slightly shorter than wide between tegulae, propodeum with border between dorsal and lateral surfaces rounded, dorsal and posterior surfaces angled. It differs from *O.altus* in the following characters: head in frontal view 1.1× as wide as high (head in frontal view 1.3× as wide as high in *O.altus*), clypeus in frontal view 1.2× as wide as high (clypeus in frontal view nearly as wide as high in *O.altus*), apical margin of clypeus shallowly emarginate with distance between teeth of clypeus about half width of clypeus between inner compound eye margins (apical margin of clypeus deeply emarginate, half-oval-shaped, distance between teeth of clypeus less than half width of clypeus between inner compound eye margins in *O.altus*).

#### Description.

**Figure 1.**
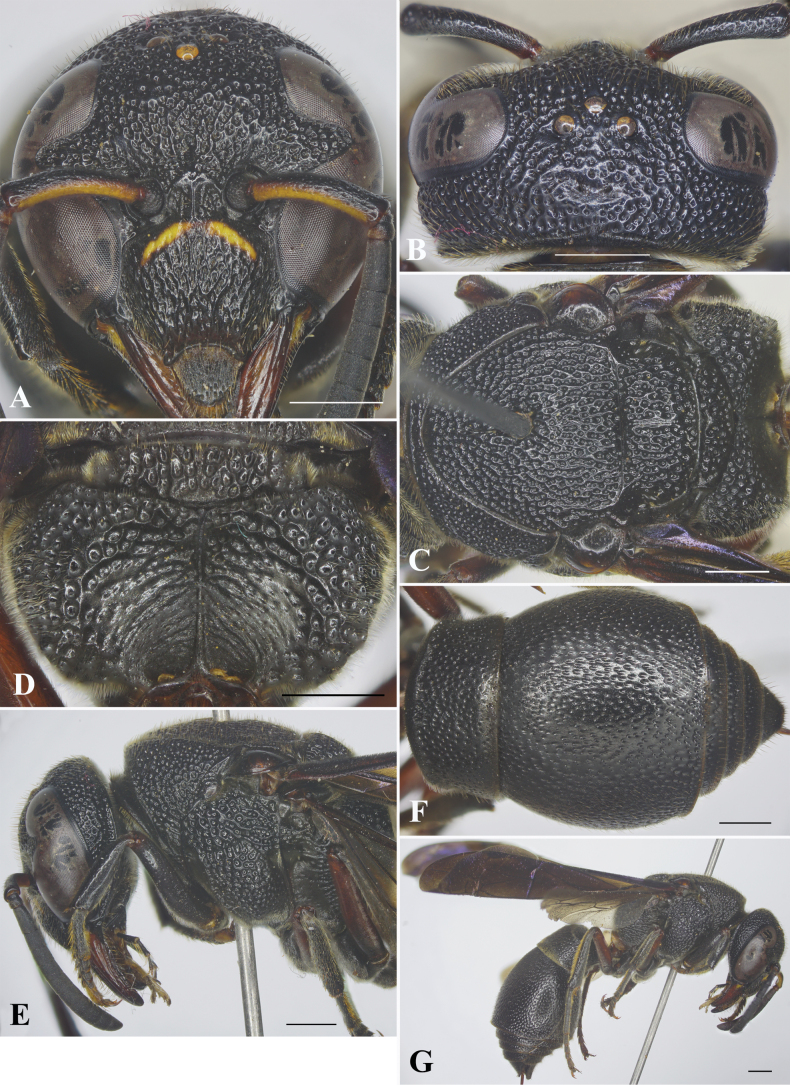
*Orancistrocerusthanghen* sp. nov., female holotype **A** frontal view **B** head, dorsal view **C** mesosoma, dorsal view **D** propodeum, posterior view **E** head and mesosoma, lateral view **F** metasoma, dorsal view **G** habitus. Scale bars: 1 mm.

**Female** (Fig. 1G): body length 13.0–14.0 mm (holotype = 13.5 mm); forewing length 12.0–13.0 mm (holotype = 12.5 mm).

***Structure*.** Head in frontal view subcircular, 1.1× as wide as high (Fig. 1A). Vertex produced behind posterior ocelli, then sloping down towards occipital carina, with cephalic foveae small, bearing dense pubescence, situated on depressed area on vertex behind posterior ocelli, and with distance between foveae slightly greater than diameter of median ocellus. Distance from lateral ocelli to apical margin of vertex 2.5× distance from lateral ocellus to inner compound eye margin (Fig. 1B). Gena in lateral view narrower than compound eye, ~0.7× as wide as compound eye; occipital carina complete, present along entire length of gena, slightly widened laterally. Inner compound eye margins in frontal view slightly convergent ventrally, in anterior view 1.1× further apart from each other at vertex than at clypeus (Fig. 1A). Clypeus in lateral view gradually convex from base to apical margin; in frontal view 1.2× as wide as high (Fig. 1A), with basal margin slightly convex medially and distinctly separated from antennal toruli; apical margin deeply emarginate medially, forming blunt tooth on each side, distance between teeth ~0.5× width of clypeus between inner compound eye margins. Mandible with prominent wide teeth, fourth tooth blunt apically. Scape long, ~4.4× as long as its maximum width, slightly curved; FI ~1.8× longer than wide, FII–III longer than wide, FIV–IX wider than long, terminal flagellomere bullet-shaped, approximately as long as its basal width. Mesosoma longer than wide in dorsal view (Fig. 1C). Pronotal carina present, rounded at humeral angle, reaching ventral corner of pronotum. Mesoscutum slightly convex, shorter than wide between tegulae, length ~0.9× width, with depressed and parallel furrows apically (depression exceptionally inconspicuous and hard to recognize in holotype and three paratypes because they fuse with large punctures), and two carinae laterally at apical one-third (Fig. 1C), with middle narrow furrow present at basal one third. Disc of mesoscutellum slightly convex, with middle furrow present throughout (Fig. 1C). Metanotum slightly convex. Propodeum (Fig. 1D) conspicuously excavated medially, with posterior surface distinctly concave, at basal half of median carina running to apical margin; border between dorsal and lateral surfaces rounded, posterior and lateral surfaces angled. TI in dorsal view narrower than TII, truncate at base (Fig. 1F); anterior vertical surface of TI slightly convex, with sparse shallow punctures, clearly separated from posterior horizontal surface by carina. TI in dorsal view ~2.2× as wide as long; TII in dorsal view ~1.2 × wider than long (Fig. 1F); SII in lateral view depressed basally, then gradually and slightly convex to apical margin.

***Sculpture*.** Clypeus with coarse punctures on disc; border between punctures raised to form longitudinal striations medially, with smaller and deeper punctures laterally. Mandible with row of punctures laterally. Frons densely covered with coarse, flat-bottomed punctures, interspaces between punctures narrow and raised to form reticulation. Vertex and gena with punctures similar to those on frons; gena with several short, transverse, conspicuous striae laterally; occipital carina slightly widened laterally. Pronotum with punctures similar to those on vertex. Mesoscutum covered with flat-bottomed punctures, punctures smaller than those on vertex and usually forming row of punctures with interspaces between rows raised to form reticulations, reticulation tending to form longitudinal carina; mesoscutellum with punctures similar to those on mesoscutum, punctures on metanotum coarser than those on mesoscutum. Mesepisternum with flat-bottomed punctures, punctures coarser and larger to those on pronotum posterodorsally, smooth anteroventrally; border between posterodorsal and anteroventral parts distinct, without epicnemial carina. Dorsal part of metapleuron largely smooth and with several punctures at upper part, ventral part largely smooth, with several sparse and shallow punctures, and some short striae laterally. Propodeum with coarse, large, flat-bottomed punctures dorsally; punctures are shallower, smaller, and sparser laterally; angle between dorsal and lateral surfaces of propodeum somewhat rounded; posteriorly surface of propodeum with punctures at upper and lateral parts, and with oblique weak carina at lower part, with a large smooth area centrally. Tegulae with sparse small punctures laterally, the remaining surface with minute punctures. Metasomal segment I covered with dense, strong and well defined punctures dorsally, sparse and fine punctures dorso-anteriorly, distance between punctures on TI narrower than puncture diameter; TII with undefined punctures, punctures shallower than those on TI, punctures near apical margins deeper, larger and coarser than those on other part of the tergum, interspaces between punctures usually equal to puncture diameter at central part; TIII–V with punctures conspicuous and deep, smaller than those on TII apically; TVI with minute punctures; SII with punctures deeper and larger than those on margins of TII laterally; SIII–V with punctures smaller and shallower than those on SII; SVI with minute punctures; SI with narrow basal part smooth.

***Color*.** Black; a narrow, curved, yellow strip near base of clypeus; yellow spots near base of mandible; antennal scape orange-yellow beneath; mandible, propodeal valvulae, and all trochanters reddish-brown. Wings brown with violet reflection; veins dark brown.

***Pubescence*.** Body with short, sparse, yellow or silver setae except lower part of propodeum with longer and denser silver setae.

**Male.** Unknown.

#### Distribution.

Central Vietnam.

#### Etymology.

The specific epithet is a noun in apposition that refers to the name of the lake where the holotype was collected.

### ﻿ Orancistrocerus thanhnhat sp. nov.

Taxon classificationAnimaliaHymenopteraVespidae

57F415B0-DF66-50FA-89D0-97BC0A3AC0E9

https://zoobank.org/B92020E8-DE78-43BF-8050-E4359442C3D2

Figs 2A–G , 3A–D

#### Material examined.

***Holotype*.** Vietnam • ♂, Cao Bang, Ha Lang, Thanh Nhat; 22°42'8"N, 106°39'49"E; alt. 332 m; 18.v.2023; Lien Thi Phuong Nguyen, Cuong Quang Nguyen & Ngat Thi Tran leg.; IEBR.

#### Diagnosis.

This species can be distinguished from other species in the genus in the following combination of characters: clypeus in frontal view as wide as high, apical margin widely emarginate medially, forming short, blunt teeth on each side, distance between teeth >½ width of clypeus between inner compound eye margins (~0.55× width of clypeus between inner compound eye margins); occipital carina conspicuously widened laterally; mesoscutum in dorsal view slightly shorter than wide between tegulae; TI in dorsal view ~2.5 × as wide as long; TII sparsely punctate, interspaces much greater (2–3×) than puncture diameter, except at base and at apical margin with interspaces smaller than puncture diameter.

The new species is similar to *O.drewseni* in that both have TII sparsely punctate centrally, interspaces much greater (2–3×) than puncture diameter. However, it differs from *O.drewseni* in the following characters: mesoscutum in dorsal view slightly shorter than wide between tegulae (mesoscutum in dorsal view longer than wide between tegulae in *O.drewseni*); TII with punctures conspicuous (TII with punctures less conspicuous in *O.drewseni*); TII not lamellate apically (TII lamellate apically in *O.drewseni*); TI–II with narrow, lateral stripes of orange-yellow (TI–II with apical thick yellow bands in *O.drewseni*); and digitus gradually narrowed from base to apex, penis valves long, more than 2× as long as basal apodeme (digitus almost parallel from base to half, then narrowed to apex in *O.drewseni* (van der Vecht 1963: fig. 8f); penis valves shorter, less than 2× as long as basal apodeme (van der Vecht 1963: fig. 8g)).

#### Description.

**Male** (Fig. 3A): body length 13.2 mm; forewing length 12.5 mm.

**Figure 2.**
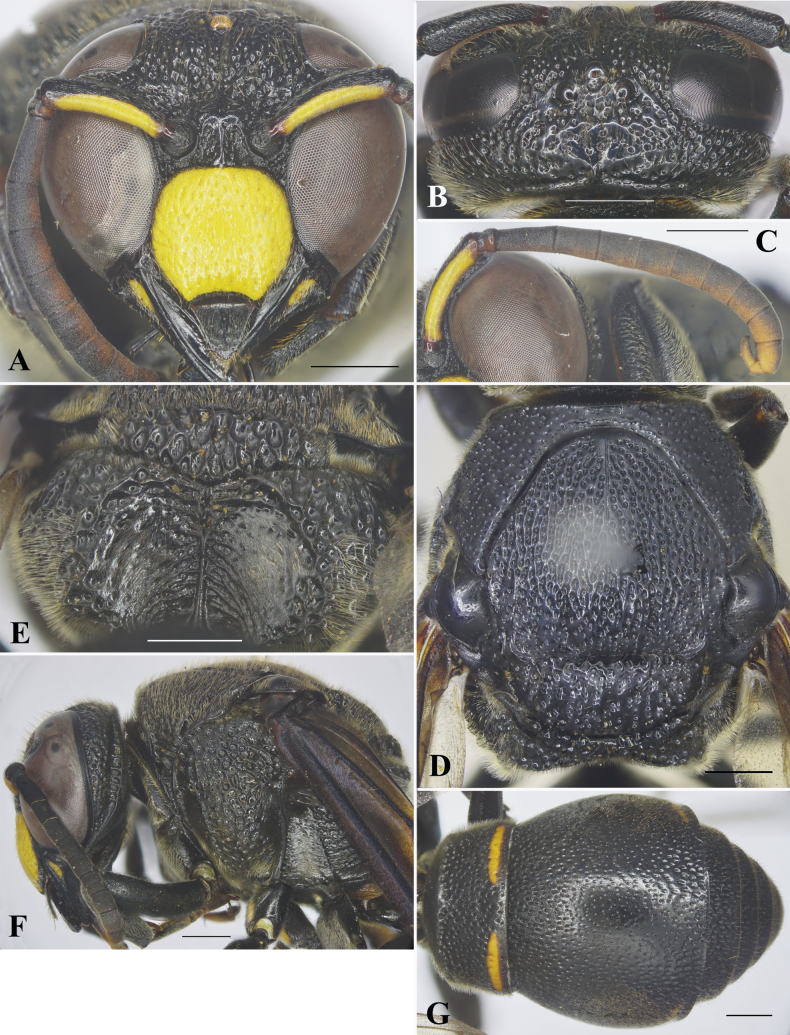
*Orancistrocerusthanhnhat* sp. nov., male holotype **A** frontal view **B** head, dorsal view **C** antenna **D** mesosoma, dorsal view **E** propodeum, posterior view **F** head and mesosoma, lateral view **G** metasoma, dorsal view. Scale bars: 1 mm.

***Structure*.** Head in frontal view subcircular, ~1.3× as wide as high (Fig. 2A). Vertex sloping down behind lateral ocelli towards occipital carina, without cephalic foveae. Distance from lateral ocelli to apical margin of vertex 2.7× distance from lateral ocellus to inner compound eye margin (Fig. 2B). Gena in lateral view much narrower than compound eye, ~0.6× as wide as compound eye; occipital carina complete, present along entire length of gena, markedly widened laterally (Fig. 2F). Inner compound eye margins in frontal view convergent ventrally, in anterior view 1.2× further apart from each other at vertex than at clypeus (Fig. 2A). Clypeus in lateral view gradually convex from base to apical margin; in frontal view as wide as high (Fig. 2A), with basal margin almost straight medially and distinctly separated from antennal toruli; apical margin widely emarginate medially, forming blunt short teeth on each side, distance between teeth >½ width of clypeus between inner compound eye margins (~0.55× width of clypeus between inner compound eye margins). Mandible with prominent wide teeth. Scape long, ~4.2× as long as its maximum width, slightly curved; FI long, ~2.3× longer than wide, FII ~1.5× as long as wide, FIII ~1.3× as long as wide, FIV slightly longer than wide, FV–VIII almost as long as wide, FIX ~1.2× longer than wide, FX small, terminal flagellomere slender, ~2.7× as long as its basal width, slightly curved (Fig. 2C). Mesosoma longer than wide in dorsal view (Fig. 2D). Pronotal carina present, rounded at humeral angle, reaching ventral corner of pronotum. Mesoscutum slightly convex, slightly shorter than wide between tegulae, with depressed and oblique furrows apically, and two carinae laterally at apical one-third (Fig. 2D) (with middle narrow furrow present at basal one-third). Disc of mesoscutellum convex, in lateral view at same level as mesoscutum (Fig. 2F), narrowly depressed basally. Metanotum slightly convex. Propodeum (Fig. 2E) conspicuously excavated medially, with posterior surface distinctly concave, at basal quarter with median carina running to apical margin; border between dorsal and lateral surfaces slightly angled, posterior and lateral surfaces angled. TI in dorsal view narrower than TII, truncate at base (Fig. 2G); anterior vertical surface of TI slightly convex, with sparse shallow punctures, clearly separated from posterior horizontal surface by carina. TI in dorsal view ~2.5× as wide as long; TII in dorsal view ~1.1× wider than long (Fig. 2G); SII in lateral view slightly depressed basally, then gradually and slightly convex to apical margin, with weak medial furrow at basal half.

***Sculpturing*.** Clypeus with sparse, shallow, large, undefined punctures on disc; border between punctures smooth. Mandible with row of punctures laterally. Frons densely covered with coarse, flat-bottomed punctures; interspaces between punctures narrow and raised to form reticulation. Vertex with coarse punctures, punctures equal in size; gena with punctures similar to those on apical half of vertex, punctures smaller and less coarse in basal half, with several short transverse conspicuous striae laterally; occipital carina markedly widened laterally (Fig. 2F). Pronotum with punctures less coarse than those on vertex. Mesoscutum covered with flat-bottomed punctures, punctures equal in size, interspaces between punctures forming reticulation that tends to form longitudinal carina centrally; mesoscutellum with punctures similar to those on pronotum, punctures on metanotum denser than those on mesoscutellum, interspaces between punctures narrow and raised to form reticulation. Mesepisternum with flat-bottomed punctures, punctures coarser and larger than those on pronotum posterodorsally, smooth anteroventrally; border between posterodorsal and anteroventral parts distinct, without epicnemial carina. Dorsal part of metapleuron largely smooth and with several short and conspicuous striae, ventral part largely smooth, with sparse and shallow punctures laterally. Propodeum with coarse, large, flat-bottomed punctures dorsally; punctures are shallower, smaller, and sparser laterally; angle between dorsal and lateral parts of propodeum somewhat rounded; posteriorly surface of propodeum with punctures at upper and lateral parts, with oblique weak carina at lower part, and with a large smooth area centrally. Tegulae with minute punctures. Metasomal segment I covered with sparse and conspicuous punctures dorsally, fine and sparse punctures dorso-anteriorly, distance between punctures greater than puncture diameter; TII with punctures shallower than those on TI, punctures near apical margins deeper, larger and coarser than those on other parts of the tergum, interspaces between punctures greater (~2–3×) than puncture diameter at central part; TIII–V with punctures conspicuous and deep, smaller than those on TII; TVI and TVII with minute punctures; SII with sparse punctures, punctures deeper and larger than those on margins of TII laterally; SIII–V with punctures smaller and shallower than those on SII; SI with narrow basal part smooth.

**Figure 3.**
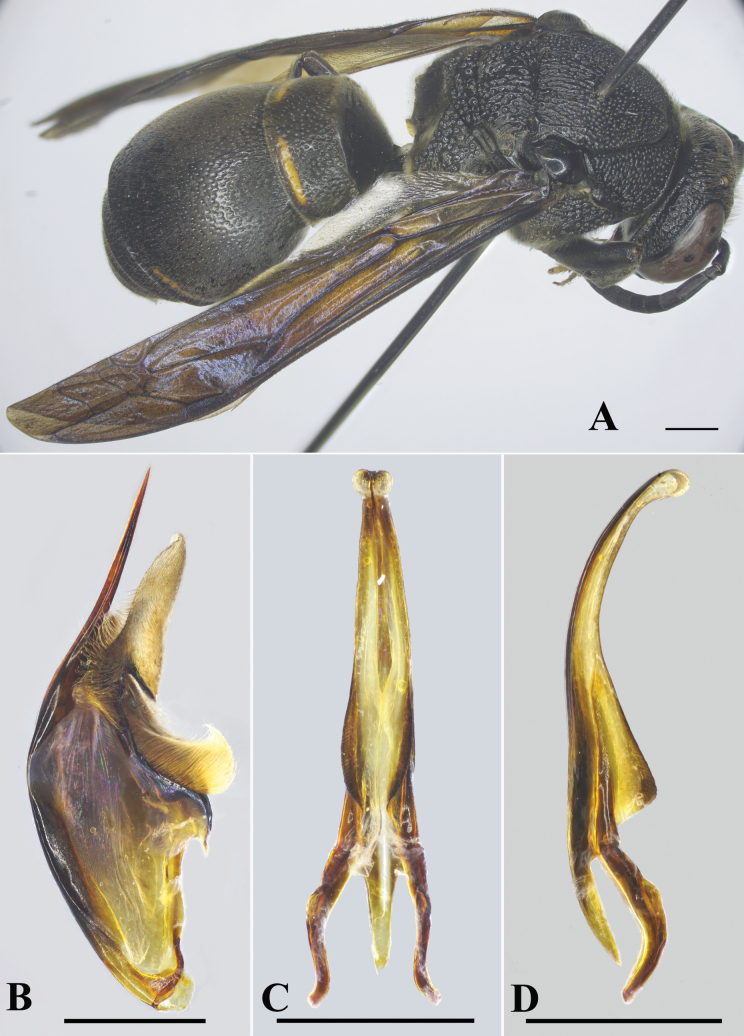
*Orancistrocerusthanhnhat* sp. nov., male holotype **A** habitus, dorsolateral view **B** genitalia, inner aspect of paramere with volsella and digitus **C** aedeagus, ventral view **D** aedeagus, lateral view. Scale bars: 1 mm.

***Color*.** Black; clypeus yellow, except for black margin; yellow spot near base of mandible; beneath scape yellow; flagellomeres beneath light brown; two short, orange-yellow bands at apical margins of TI and TII. Propodeal valvulae dark brown. Middle coxae with two short, yellow strips; hind coxae with a yellow spot. Wings brown, transparent; veins dark brown (Fig. 3A).

***Pubescence*.** Head and thorax with long, dense, yellow setae; metasoma with short, dense, yellow setae.

**Female.** Unknown.

***Genitalia*.** As in Fig. 3B–D. Parameral spine lacking setae. Volsella flattened, spatulate, wide on inner aspect, and without setae apically. Digitus gradually narrowing from base to apex, all setose (Fig. 3B). Penis valve long, ~2.2× as long as basal apodeme (Fig. 3C), in profile apical part conspicuously produced into triangular lobe (Fig. 3D).

#### Distribution.

Northern Vietnam.

#### Etymology.

The specific epithet is a noun in apposition that refers to the name of the town where the holotype was collected.

### ﻿ Orancistrocerus aterrimus

Taxon classificationAnimaliaHymenopteraVespidae

(de Saussure, 1852)

05ABF654-40E4-5B9D-9D2A-0EE394EC841D

Fig. 4A–C

Odynerus aterrimus de Saussure, 1852: 121 (key), 128.
Orancistrocerus aterrimus (de Saussure); Truong et al. 2019: 599 (key).

#### Material examined.

Vietnam • 1♀; Ha Giang, Dong Van; 12 July 2015; Khuat Dang Long; • 2♀; Cao Bang, Nguyen Binh, Tam Kim; 22°36'17"N, 106°01'47.6"E; alt. 299 m; 18.x. 2015; • 1♀; Cao Bang, Nguyen Binh, Tran Hung Dao forest; 22°36'17"N, 106°01'47.6"E; alt. 470 m; 18.x. 2015; Nguyen Thi Phuong Lien, Nguyen Dac Dai & Nguyen Phuong Minh leg.; • 1♀; Cao Bang, Thanh Nhat, Ha Lang; 22°42'9"N, 106°39'52"E, alt. 250 m; 19.xi.2023; Nguyen Quang Cuong & Tran Thi Ngat leg.; • 2♀♀; Cao Bang, Tra Linh, Thang Hen Lake; 22°45'33"N, 106°17'46"E; alt. 578 m; 4.viii.2022; Nguyen Thi Phuong Lien et al. leg.; • 2♀♀; Cao Bang, Tra Linh, Thang Hen Lake; 22°45'33"N, 106°17'46"E; alt. 550 m; 18.ix.2023; Nguyen Thi Phuong Lien, Tran Thi Ngat, Nguyen Duc Anh leg.; • 4♀♀, Cao Bang, Trung Khanh, Dam Thuy, Nguom Ngao cave; 22°50'43.2"N, 106°22.2"E; 19.ix.2023; Nguyen Thi Phuong Lien, Tran Thi Ngat leg.; • 1♀; Bac Kan, Cho Don, Binh Trai, Nam Xuan Lac NP; 22°16'65"N, 108°11'08"E; alt. 780 m, 12.viii.2020; Nguyen Thi Phuong Lien et al. leg.; • 1♀; Lao Cai, Sa Pa, Ban Ho, Hoang Lien NP; 27–29.vii.2008; Nguyen Thi Phuong Lien leg.; • 1♀; Lao Cai, Bat Xat, Y Ty, Sim San, 22°37'48"N, 103°34'52"E; alt. 1324 m; 2.viii.2019; Nguyen Thi Phuong Lien, Nguyen Quang Cuong, Tran Thi Ngat leg.; • 1♀; Dien Bien, Muong Fang; alt. 500 m; 23.vii.2009; Nguyen Thi Phuong Lien, Pham Huy Phong, Kojima Junichi leg.; • 1♀; Tuyen Quang, Na Hang, Na Hang NR, Son Phu ranger station; 22°21.2'07"N, 105°24'34.7"E; alt. 264 m, 10.vi.2015, Nguyen Thi Phuong Lien, Nguyen Dac Dai, Truong Xuan Lam leg.; • 1♂; Lang Son, Huu Lung, Cai Kinh; 20°31'37.6"N, 105°0'24.2"E; alt. 86 m, 16.vii.2016; Nguyen Thi Phuong Lien, Nguyen Dac Dai, Tran Thi Ngat leg.; • 1♀; Lang Son, Huu Lung, Huu Lien, Lan Nghe, Huu Lien NR; 21°33'48.6"N, 106°24'36.4"E; alt. 289 m; 11.vi.2018; Nguyen Thi Phuong Lien et al. leg.; • 1♀; Bac Giang, Son Dong, An Lac, Dong Bay; 21°20'42.8"N, 106°56'31.1"E; 12.viii.2012; Kojima Junichi, Nugroho Hari, Nguyen Thi Phuong Lien leg.; • 1♀; Quang Ninh, Hoanh Bo, Dong Quang; 2.viii.2013; Tran Van Tuan leg.; • 1♀; Hai Phong, Cat Hai, Cat Ba NP, 20°47'38"N, 106°59'45"E; 25.vii.2013; Nguyen Thi Phuong Lien, Nguyen Dac Dai leg.; • 1♀; Vinh Phuc, Ngoc Thanh, Me Linh; 7.vi.2001; Truong Xuan Lam leg; • 4♀♀; Ha Noi, Ba Trai, Ba Vi; 9.vii.2017; Nguyen Thi Phuong Lien, Luong Viet Tuan leg.; • 1♀; Hoa Binh, Bao Hieu, Yen Thuy; 14.vii.1999; Truong Xuan Lam leg.; 1♀; Hoa Binh, Yen Thuy; 1.v.2012; Hoang Vu Tru leg.; • 1♂; Hoa Binh, Mai Chau, Chieng Chau, Lac village; alt. 600 m; 1.vi.2008; Nguyen Thi Phuong Lien leg.; • 1♀, 1♂; Thanh Hoa, Thuong Xuan, Van Xuan, Hon Can, Xuan Lien NP; 19°51'55.5"N, 105°14'28.8"E; alt. 120 m; Nest#2012-TH-Eumeninae-01; 23.viii.2012; Nguyen Thi Phuong Lien leg.; • 1♀; Thanh Hoa, Thuong Xuan, Van Xuan, Hon Can, Xuan Lien NP; 19°52'27.5"N, 105°14'20.8"E; alt. 110 m; 23.viii.2012; Nguyen Thi Phuong Lien leg.; • 1♀; Da Nang, Vinh Cuu, Phu Ly, Vinh An; 30.vii.2008; ISD-c leg.; • 1♀; Dak Lak, Krong Bong, Krong Kmar, Chu Yang Sin NP; 12°25'02.8"N, 108°22'30.8"E; alt. 1081 m; 4.v.2016; Nguyen Thi Phuong Lien, Nguyen Dac Dai, Tran Thi Ngat leg.; • 2♀♀; Lam Dong, Da Lat, Bidoup Nui Ba NP; 12°10'56.7"N, 108°40'47.9"E; alt. 1458 m; 7.v.2016; Nguyen Thi Phuong Lien, Nguyen Dac Dai, Tran Thi Ngat leg.; IEBR. CHINA: • 1♀, 1♂; Tianzhu, Guizhou, viii. 2009; Wang Yang-Wen leg.; IEBR.

**Figure 4.**
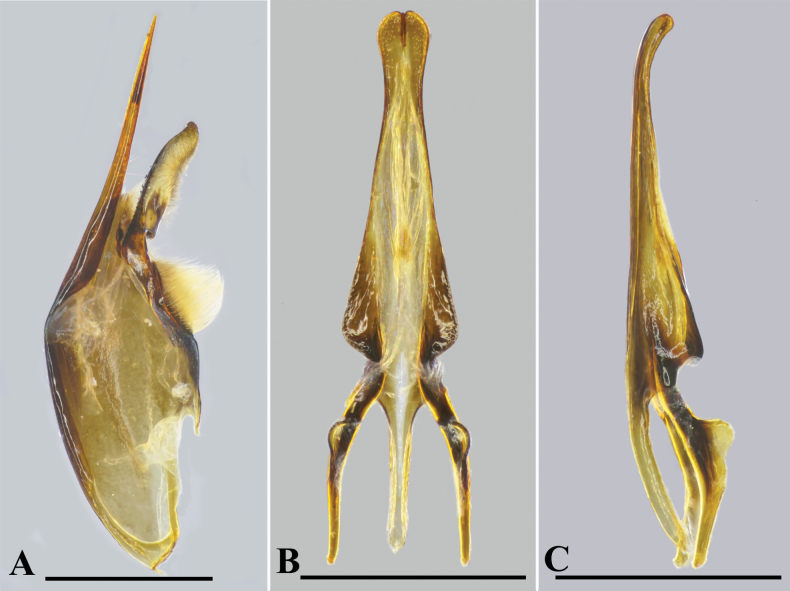
*Orancistrocerusaterrimus*, male **A** genitalia, inner aspect of paramere with volsella and digitus **B** aedeagus, ventral view **C** aedeagus, lateral view. Scale bars: 1 mm.

***Male genitalia*.** As in Fig. 4A–C. Parameral spine without setae. Volsella flattened, spatulate, wide on inner aspect, without setae at top. Digitus almost parallel from near base to near apex, then gradually narrowed to apex (Fig. 4A), its surface covered with setae except glabrous at base, its inner lateral margin with some small and short tubercles at middle, serrated at base. Penis valve short, ~1.7× as long as basal apodeme (Fig. 4B), in profile apical part markedly produced into triangular lobe (Fig. 4C).

#### Remarks.

Four subspecies have been recognized in *O.aterrimus*: *O.a.aterrimus* from China and Vietnam; *O.a.erythropus* (Bingham, 1897) from India, Myanmar, Laos, and Thailand; *O.a.khasianus* (Cameron, 1900) from India; and *O.a.nigriceps* van der Vecht, 1963 from China and Vietnam (van der Vecht 1963; Kumar and Sharma 2012; Selis 2018; Truong et al. 2019).

In Vietnam, *O.a.aterrimus* has been recorded in Da Nang province by Selis (2018). *Orancistrocerusa.nigriceps* has been recorded from Phu Quoc Island, Kien Giang province by van der Vecht (1963) and Truong et al. (2019). Having carefully examined specimens studied by Truong et al. (2019), we found that almost all specimens identified as *O.a.nigriceps* in their paper are actually *O.a.aterrimus*, with color of body and wing fitting well into the description by van der Vecht (1963): wings brown with violaceous effulgence, body black, a small spot on mandibles, two small inter-antennal spots, and a line at underside of scape yellow. Two males and two females have the wing base more or less clear hyaline, as the color character to separate *O.a.erythropus* from *O.a.aterrimus* as noted by van der Vecht (1963). Of those, one male from Xuan Lien NP, Thanh Hoa province has its wings fully clear hyaline, and this male comes from the same nest (Nest #2012-TH-Eumeninae-01) as a female, but the female is colored as *O.a.aterrimus* (wings brown with violaceous effulgence). Thus, the color of wing base cannot be used to separate the subspecies *O.a.aterrimus* and *O.a.erythropus*, and, thus, we synonymize *O.a.erythropus* with the nominate subspecies (new synonymy). One specimen examined by Truong et al. (2019) has subhyaline wings, but the clypeus and antennal scape were not entirely black (clypeus black with two large yellow spots laterally and one small yellow spot medially; antennal scape yellow beneath), and we cannot assign this specimen to any subspecies of *O.aterrimus*. At the moment, two subspecies of *O.aterrimus* are recorded in Vietnam, namely *O.a.aterrimus* and *O.a.nigriceps*. Due to the lack of specimens, the status is left as it is until more specimens are available to us. In this paper, all specimens examined are treated at the species level. Herein, the male genitalia of the species are described and figured for the first time (based on a male of *O.a.aterrimus*).

### ﻿ Orancistrocerus altus

Taxon classificationAnimaliaHymenopteraVespidae

Truong, Bozdoğan & Nguyen, 2019

1046EB60-F855-5010-AFC9-4DD9A526E285

Orancistrocerus altus Truong, Bozdoğan & Nguyen, 2019: 596.

#### Remarks.

The species is known only from the type locality, Huu Lien Nature Reserve, Lang Son Province, northern Vietnam.

### ﻿Key to species of *Orancistrocerus*

This is an updated key based on that of Truong et al. (2019). The characters are applicable to both sexes unless specified.

**Table:** 

1	TII sparsely punctate, interspaces much greater (2–3×) than puncture diameter (Fig. 2G), except at base and at apical margin with interspaces smaller than puncture diameter; mesosoma not colored as below	**2**
–	TII densely punctate, interspaces larger (~1.5×) than puncture diameter (Fig. 1F), except at base and at apical margin with interspaces equal or smaller than puncture diameter; mesosoma with yellow apical band on TI–II or extensively marked with red or entirely black	**3**
2	Mesoscutum in dorsal view slightly shorter than wide between tegulae; TII not lamellate apically; punctures on TII conspicuous; digitus gradually narrowed from base to apex (Fig. 3B), penis valve long, more than 2× as long as basal apodeme (Fig. 3C); spot near base of mandibles and beneath scape yellow; lateral strips on TI–II short and narrowly orange-yellow	***O.thanhnhat* sp. nov.**
–	Mesoscutum in dorsal view longer than wide between tegulae; TII lamellate apically; punctures on TII less conspicuous; digitus almost parallel from base to midlength, then narrowing to apex (van der Vecht 1963: fig. 8f), penis valve shorter, less than 2× as long as basal apodeme (van der Vecht 1963: fig. 8g); spot near base of mandibles, beneath scape, and large inter-antennal spot orange-yellow; apical bands of TI–II wide, orange-yellow	***O.drewseni* (de Saussure, 1857)**
3	Female clypeus with apical margin deeply emarginate, forming two sharp teeth laterally (Truong et al. 2019: fig. 3; Fig. 1A)	**4**
–	Female clypeus with apical margin shallowly emarginate, forming two blunt teeth laterally (Truong et al. 2019: fig. 1)	**5**
4	Female clypeal emargination exceedingly deep, half-oval shaped (Truong et al. 2019: fig. 3), with an inverse U-shaped mark near basal margin of clypeus and apical bands of TI–II yellow	***O.altus* Truong, Bozdogan & Nguyen, 2019**
–	Female clypeal emargination shallower (Fig. 1A), with a narrow, curved, yellow strip near base of clypeus, metasoma all black	***O.thanghen* sp. nov.**
5	Wings yellowish or light cupreous brown, not conspicuously infuscate in apical half; mesosoma extensively marked with red	***O.moelleri* (Bingham, 1897)**
–	Wings subhyaline or yellow, conspicuously infuscate at apex; mesosoma not colored as above	**6**
6	Mandible with ivory white spot at base; basal part of wings rich golden hyaline, apical half fuscous with golden and violaceous reflections	***O.aterrimus* (de Saussure,1852)**
–	Mandibles with yellow spot at base; wings with less pronounced yellow tinge, apical cloud covers marginal cell, second and third submarginal cells and areas below and beyond these cells	***O.bicoloripennis* (Gribodo, 1892)**

## Supplementary Material

XML Treatment for Orancistrocerus

XML Treatment for Orancistrocerus thanghen

XML Treatment for Orancistrocerus thanhnhat

XML Treatment for Orancistrocerus aterrimus

XML Treatment for Orancistrocerus altus

## ﻿Additional information

### Author ORCIDs

Lien Thi Phuong Nguyen https://orcid.org/0000-0003-3527-9577

Anh D. Nguyen https://orcid.org/0000-0001-9273-0040

Hoa T. Dang https://orcid.org/0000-0002-8312-2825

### Data availability

All of the data that support the findings of this study are available in the main text.
